# Tailoring Lattice Strain and Ferroelectric Polarization of Epitaxial BaTiO_3_ Thin Films on Si(001)

**DOI:** 10.1038/s41598-017-18842-5

**Published:** 2018-01-11

**Authors:** Jike Lyu, Ignasi Fina, Raul Solanas, Josep Fontcuberta, Florencio Sánchez

**Affiliations:** Institut de Ciència de Materials de Barcelona (ICMAB-CSIC), Campus UAB, Bellaterra, 08193 Barcelona, Spain

## Abstract

Ferroelectric BaTiO_3_ films with large polarization have been integrated with Si(001) by pulsed laser deposition. High quality *c*-oriented epitaxial films are obtained in a substrate temperature range of about 300 °C wide. The deposition temperature critically affects the growth kinetics and thermodynamics balance, resulting on a high impact in the strain of the BaTiO_3_ polar axis, which can exceed 2% in films thicker than 100 nm. The ferroelectric polarization scales with the strain and therefore deposition temperature can be used as an efficient tool to tailor ferroelectric polarization. The developed strategy overcomes the main limitations of the conventional strain engineering methodologies based on substrate selection: it can be applied to films on specific substrates including Si(001) and perovskites, and it is not restricted to ultrathin films.

## Introduction

Lattice strain generally causes dramatic effects on the properties of ferroelectrics. In particular, epitaxial strain has permitted a notable enhancement of the ferroelectric polarization and the Curie temperature^1,2^. However, conventional substrate-based strain engineering is restricted to relatively small ranges of strain and film thickness due to plastic relaxation^3–5^. Moreover, it is based on the selection of a specific substrate, whereas most applications require integration with silicon. Therefore, alternatives to the usual substrate engineering are needed, and some unconventional methods have been already developed^6^. For example, the inclusion of a secondary phase that segregates in nanocolumns in a ferroelectric BaTiO_3_ (BTO) matrix and strains the latter can be used^7^. Without using secondary phases, residual stress, which is usual in BTO films deposited by energetic techniques like pulsed laser deposition or sputtering, can be used to modify BTO lattice parameters^8–14^. The resulting strain influences severely the ferroelectric properties and highly enhanced tetragonality (c/a > 1.1) and ferroelectric remnant polarization (>50 μC/cm^2^) has been reported in BTO films on SrTiO_3_(001)^9^. The control of strain and ferroelectric polarization by thin film deposition parameters would be a versatile strategy, alternative to the classic strain engineering, that could permit controlling strain and properties of ferroelectric films integrated with silicon.

It has been reported the dependence of lattice strain on laser fluence used to grow BTO films by pulsed laser deposition on GdScO_3_(110)^8^. The films showed differences in the Curie temperature although similar polarization, and it was proposed the existence of dipole defects in the films^8^. We present a different strategy of controlling the strain in BTO films, which generates a scalable switchable polarization with strain. The strategy is based on controlling the defects that generate strain by tuning the balance between thermodynamics and kinetics in the growth of BTO via modification of substrate deposition temperature. We focus on thin films integrated with Si(001)^15–17^, and we show that epitaxial growth can be achieved in a broad deposition temperature window with a huge impact on the BTO tetragonality and ferroelectric polarization. In particular, high-quality *c*-oriented epitaxial BTO films are grown in a temperature window about 300 °C wide, permitting fine tuning of the *c*-axis (the polar axis) strain from 0% (high deposition temperature, favoring thermodynamics) to more than 2% (low deposition temperature, imposing kinetic limitations), and with the remnant polarization scaling with the c-axis. The growth strategy has permitted an unprecedented level of control of structural and ferroelectric properties of epitaxial BTO thin films on silicon, and also allows tailoring the strain in ferroelectric films on perovskite substrates.

## Results

**Figure 1:**
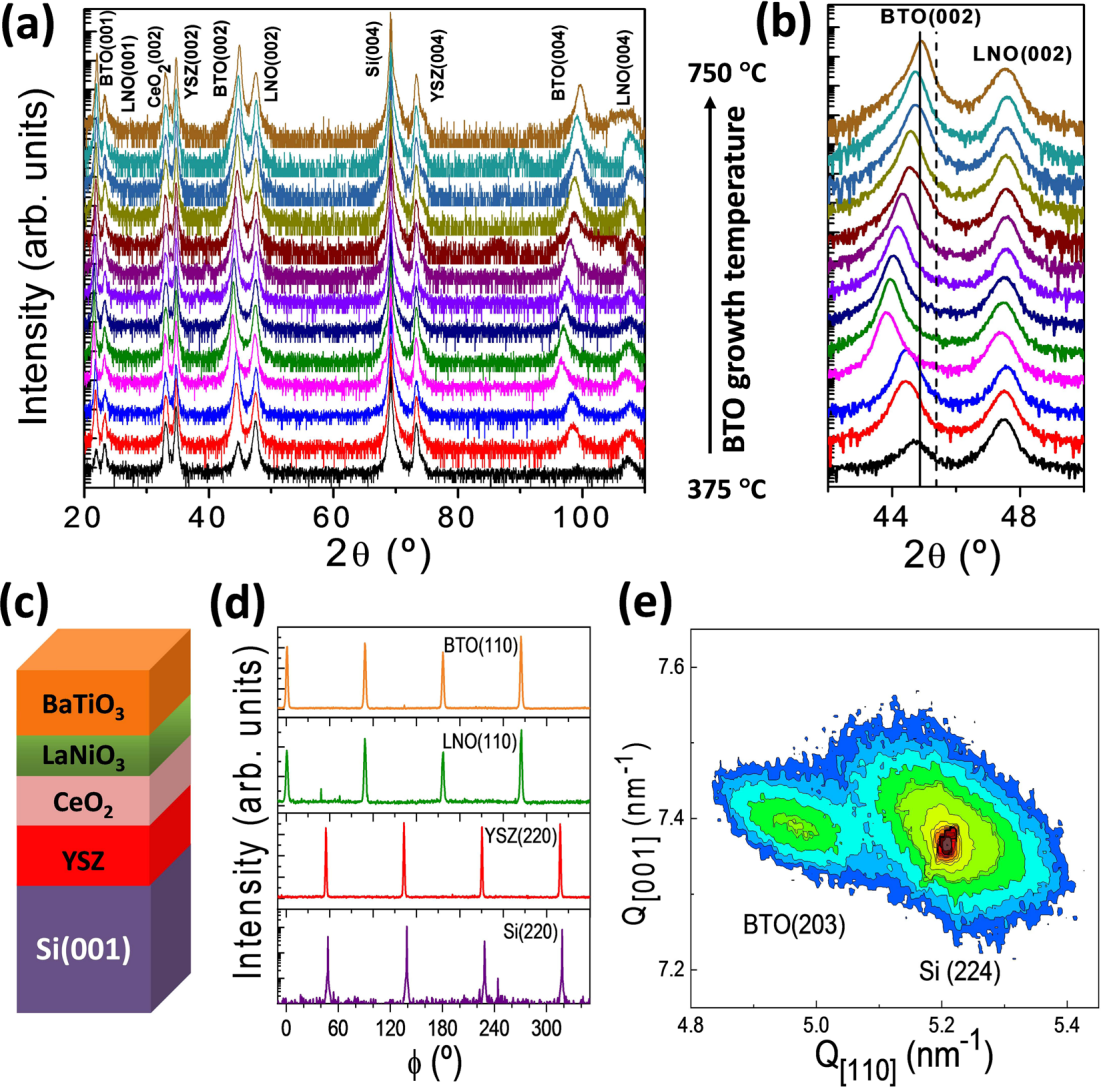
(**a**) XRD θ-2θ scans of the BTO/LNO/CeO_2_/YSZ/Si(001) series. The intensity is plotted in logarithmic scale, and the diffractograms are shifted vertically for clarity, from the T_s_ = 375 °C to the 700 °C sample. (**b**) Zoomed region of the θ-2θ scans around the (002) reflections of BTO and LNO. The vertical solid and dashed lines mark the position of the (002) and (200) reflections in bulk BTO, respectively. (**c**) Sketch of the heterostructure. (**d**) ϕ-scans around the BTO(110), LNO(110), YSZ(220) and Si(220) reflections of the T_s_ = 675 °C sample. (**e**) Reciprocal space map around the BTO(203) and Si(224) reflections of the T_s_ = 700 °C sample.

**Figure 2:**
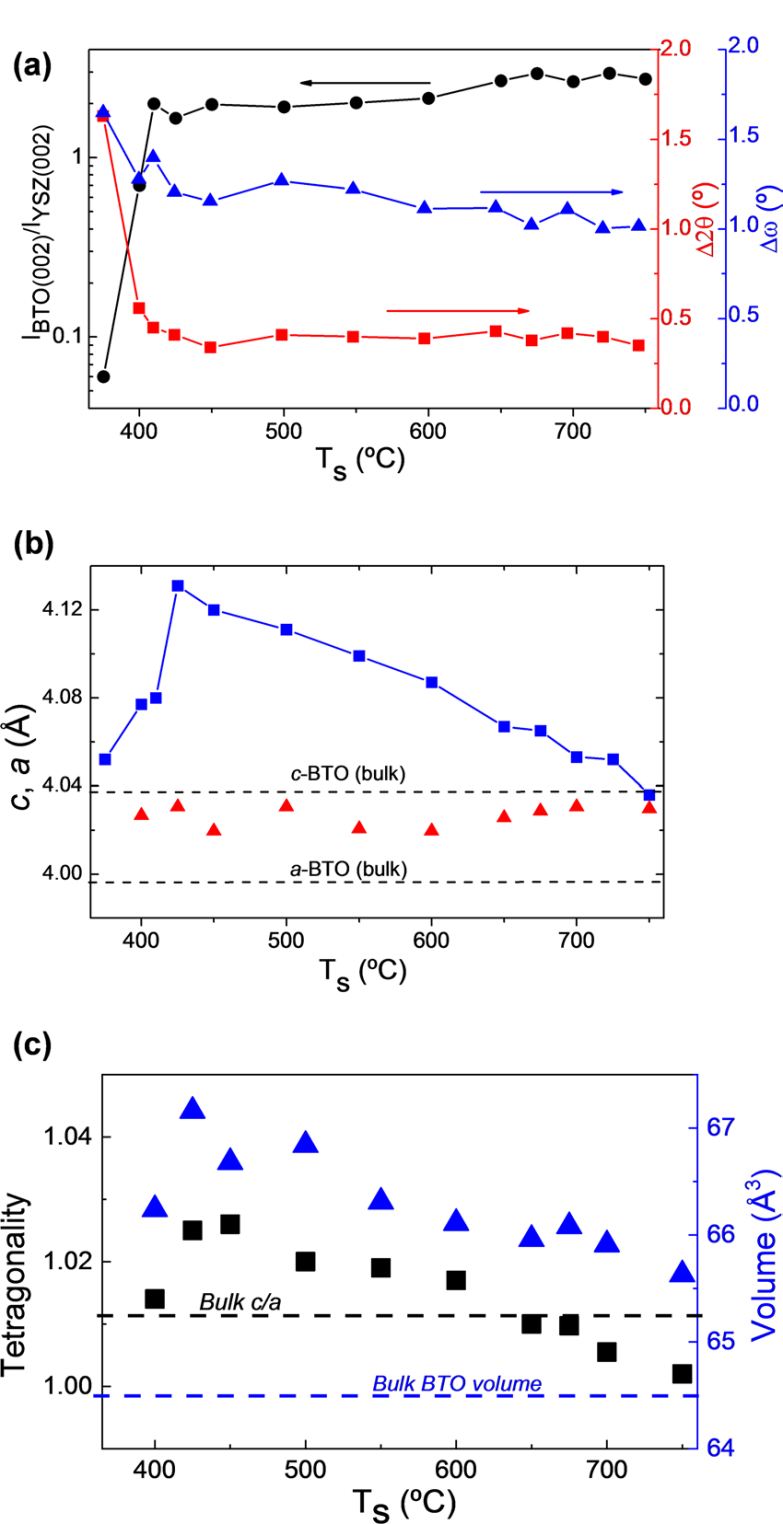
Dependence with the deposition temperature of BTO on LNO/CeO_2_/YSZ/Si(001) of (**a**) intensity ratio between the BTO(002) and YSZ(002) peaks (black circles, left axis), and full width at half maximum of the 2θ- scan (red squares, right axis), and ω-scan (blue triangles, right axis) of the BTO(002) reflection. (**b**) Dependence of the out-of-plane lattice parameter (blue squares), and in-plane lattice parameter for selected samples (red triangles). The horizontal dashed lines indicate the *a*- and *c*-axes length in bulk BTO. (**c**) Unit cell tetragonality (left axis, black squares) and volume (right axis, blue triangles). The horizontal dashed lines indicate the c/a tetragonality and unit cell volume of bulk BTO.

The specular XRD θ-2θ scans of the samples on Si(001) are shown in Fig. 1a. There are (00l) reflections from the Si substrate, YSZ, CeO_2_ and LaNiO_3_ (LNO) buffer layers, and BTO film, without peaks from spurious phases or other crystal orientations. It is remarkable that in spite of the broad range of BTO growth temperature T_s_ (close to 400 °C, from T_s_ = 375 °C to 750 °C), BTO is single (00l) oriented in all the samples. A zoomed region of the θ-2θ scans around the (002) reflections of BTO and LNO is in Fig. 1b. The solid and dashed vertical lines mark the position of the (002) and (200) reflections of bulk BTO, respectively. The intensity of the BTO(002) peak in the T_s_ = 375 °C sample, the lowest BTO growth temperature, is much reduced in comparison with the other samples. To quantify the BTO crystallization dependence on deposition temperature, the BTO(002) peak intensity has been normalized to that of YSZ(002) peak of the corresponding sample. The dependence with T_s_ of the intensity ratio, in logarithmic scale, is plotted in Fig. 2a. The intensity ratio increases sharply up to around 425 °C, with much lower dependence for higher temperatures. It indicates that the threshold temperature for BTO crystallization by pulsed laser deposition on the used buffer layers is around 375 °C. The dependences with T_s_ (Fig. 2a, right axis) of the rocking curve (Δω, ω-scan) and particularly the width (Δ2θ, θ-2θ scan) of the BTO(002) reflection also reflect the onset temperature of crystallization. Moreover, the data do not indicate differences in crystal quality between the films deposited at temperature above 425 °C, with similar values of Δω (~1.1°) and Δ2θ (~0.4°).

The position of the BTO peak depends on T_s_, being coincident with the bulk BTO(002) in the film grown at the highest temperature (T_s_ = 750 °C) and at lower 2θ angles in the other samples. This result indicates that the BTO films are c-oriented, the *c*-axis strain depending on the substrate temperature. ϕ-scans and reciprocal space maps around asymmetrical reflections confirmed epitaxial growth. A sketch of the heterostructure is presented in Fig. 1c. Figure 1d shows ϕ-scans around BTO(110), LNO(110), YSZ(220) and Si(220) reflections of the T_s_ = 675 °C sample. Each ϕ-scan shows a set of four peaks, the BTO and LNO peaks being shifted 45° with respect to the YSZ and Si ones. The lattice parameter of CeO_2_ is almost coincident with Si, and the CeO_2_(220) reflections overlap with the high intensity Si substrate peaks. It is concluded that the four layers are epitaxial, with cube-on-cube epitaxial relationship of CeO_2_ and YSZ with Si(001), whereas BTO and LNO present an in-plane rotation of 45°^15^. Reciprocal space maps around the BTO(203) and Si(224) reflections of several samples were measured (the corresponding to the T_s_ = 700 °C sample is in Fig. 1e). The BTO peak indicates homogeneous strain in all the measured samples (Supplementary Information Fig. S1). The in-plane lattice parameter is plotted (red triangles) against T_s_ in Fig. 2b, together with the out-of-plane lattice parameter (blue squares, determined from the specular θ-2θ scans) of all BTO films on Si. The horizontal dashed lines indicate the values of the *a* (3.994 Å) and c (4.038 Å) parameters of bulk BTO. It is observed that the out-of-plane parameter increases first up to a strain ε_[001]_ of 2.3% in the T_s_ = 425 °C sample, and for higher deposition temperatures decreases monotonically reaching the bulk value of the c-axis in the T_s_ = 750 °C sample. The strain displays minor variations if the films are either cooled down under high oxygen pressure (200 mbar instead of 0.2 mbar) or by additional annealing step (1 hour, 600 °C, 200 mbar). The strain and ferroelectric polarization data of these samples are presented in Supplementary Information S2. The effect of *ex-situ* annealing (1 hour, 200 mbar) on the lattice parameter *c* of the T_s_ = 450 °C and 600 °C samples was investigated too (Supplementary Information S3). The samples show almost negligible differences after annealing at 450 °C. The annealing at 600 °C has very small effect on the T_s_ = 600 °C sample, and in the T_s_ = 450 °C sample there is a small decrease of the out-of-plane parameter. Fig. S3c shows the small differences in the out-of-plane lattice parameter, including the data corresponding to the *in-situ* and *ex-situ* annealing, proving the limited effects of annealing and signaling the high stability of the films.

The in-plane parameter of the BTO films deposited at different T_s_ varies slightly between the samples, with values in the 4.021–4.032 Å range. Three factors can contribute to the expansion of the in-plane parameter: i) the epitaxial strain (it is not expected to be relevant due to the relatively large film thickness and lattice mismatch, anticipating a fast in-plane relaxation); ii) the point defects in the films (more relevant for the samples deposited at low T_s_, although the unit cell is expected to expand mainly along the out-of-plane direction due to the epitaxial in-plane matching); and iii) the higher thermal expansion coefficients of BTO than of silicon, must induce a tensile stress when the films are cooled down to room temperature. The thermal mismatch stress is more important in the high T_s_ samples. The combined effect of these factors results in similar in-plane parameter in the T_s_ = 400–750 °C range. The unit cell tetragonality (c/a) and volume (Fig. 2c) increase with T_s_ up to 450 °C and 500 °C, respectively, and for higher T_s_ both decrease monotonically. The tetragonality is smaller than in bulk BTO in the samples deposited at T_s_ higher than 650 °C. In contrast, the unit cell is expanded in all the samples, with high volume expansions ranging from around 1.9% (750 °C sample) to above 3.7% (500 °C sample).

**Figure 3:**
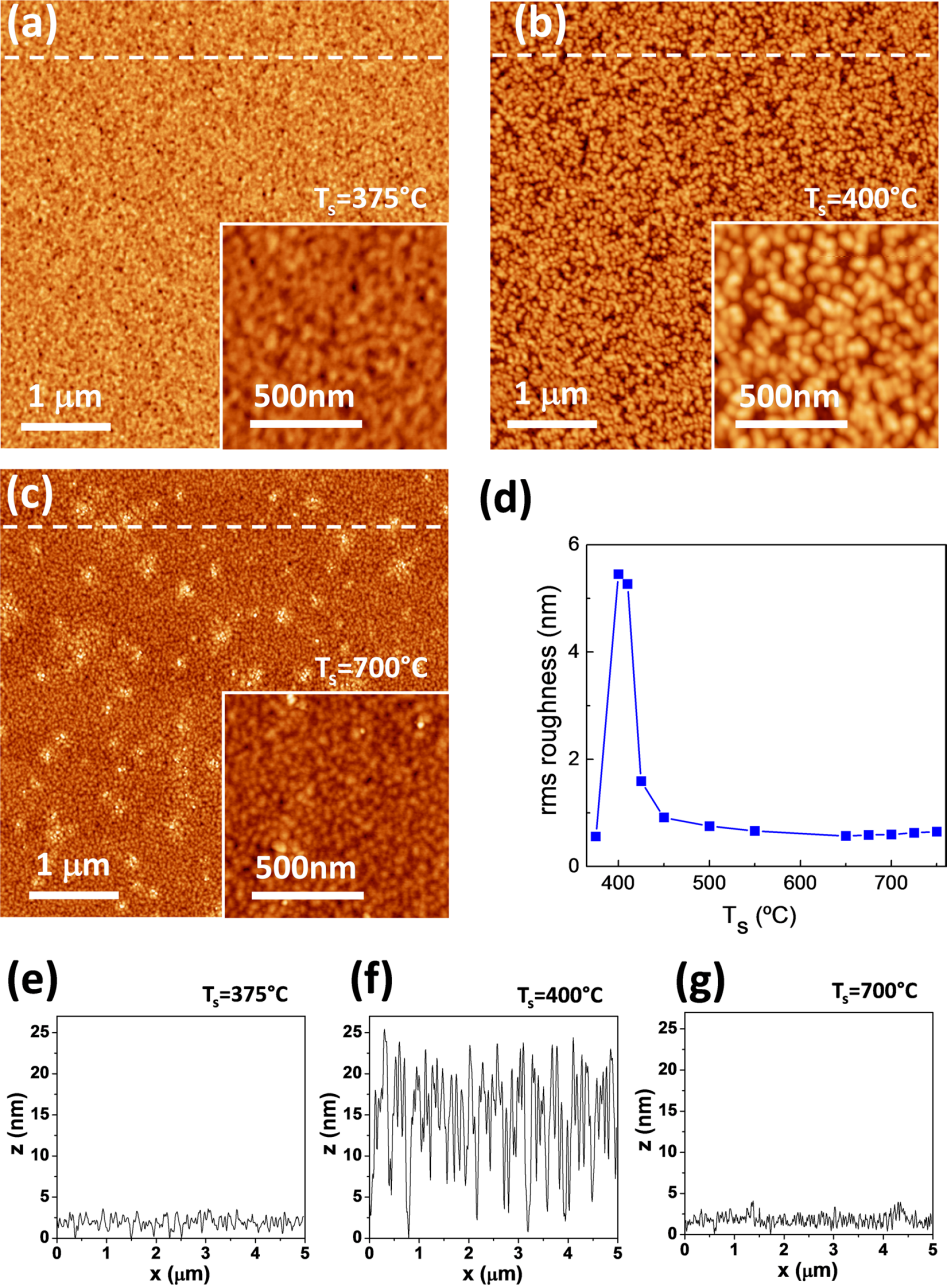
Topographic AFM images, 5 μm × 5 μm in size (inset: 1 μm × 1 μm) of the (**a**) T_s_ = 375 °C (z-scale: 7 nm), (**b**) T_s_ = 400 °C (z-scale: 40 nm), and (**c**) T_s_ = 700 °C (z-scale: 7 nm) BTO films on LNO/CeO_2_/YSZ/Si(001). Height profiles along the horizontal dashed lines shown in each 5 μm × 5 μm image are in (**e**), (**f**) and (**g**), respectively. (**d**) rms roughness as a function of T_s_.

Topographic AFM images, 5 μm × 5 μm in size, of the T_s_ = 375, 400, and 700 °C samples on Si are shown in Fig. 3a–c, respectively, with 1 μm × 1 μm images in the corresponding insets. The three images (and the corresponding ones to other films in the series) show islands with lateral size of a few tens of nm. The lateral size is around 40 nm in the T_s_ = 375 °C and 700 °C samples, and around 80 nm in the T_s_ = 400 °C sample. The morphology of the T_s_ = 375 °C sample is very homogeneous, whereas in the T_s_ = 700 °C sample there is agglomeration of a few islands of higher height in some areas. The morphology of the T_s_ = 400 and the 410 °C samples (the AFM image of the latter not shown here) differs with respect to the other samples, with flat areas between the islands, which are notably larger in height than in the other samples. Notice in Fig. 3(a–c) the differences in the z-scales. Indeed, the z-scale ranges in the topographic images are 7 nm in Fig. 3a,c, and 40 nm in Fig. 3b. The corresponding height profiles in Fig. 3e–g along the dashed lines shown in Fig. 3a–c, evidence the huge difference in islands height. The root means square (rms) roughness of all the samples on Si, calculated from 1 μm × 1 μm areas, is plotted as a function of T_s_ in Fig. 3d. There is a sharp increase from 0.56 nm to more than 5 nm as T_s_ increases from 375 to 400 °C. The rms roughness, similarly high in the T_s_ = 410 °C sample, decreases to around 1.6 nm and 0.92 nm in the T_s_ = 425 and 450 °C samples, respectively, and is below 0.75 nm for the samples grown at higher temperatures. The peaky dependence of the roughness with deposition temperature is a signature of the onset of BTO crystallization above 375 °C, being the high roughness in the 400–425 °C samples likely due to the coexistence of well crystallized islands with other regions presenting lower crystal order or amorphous state. The (001) orientation in the T_s_ = 400 °C sample, with in-plane epitaxial order confirmed by reciprocal space mapping (Supplementary Information Fig. S1) points to epitaxial columnar growth coexisting with not-epitaxial regions. On the other hand, the low surface roughness of the T_s_ = 375 °C sample indicates that surface diffusivity at this temperature is low for epitaxial growth but high enough for surface smoothing.

**Figure 4:**
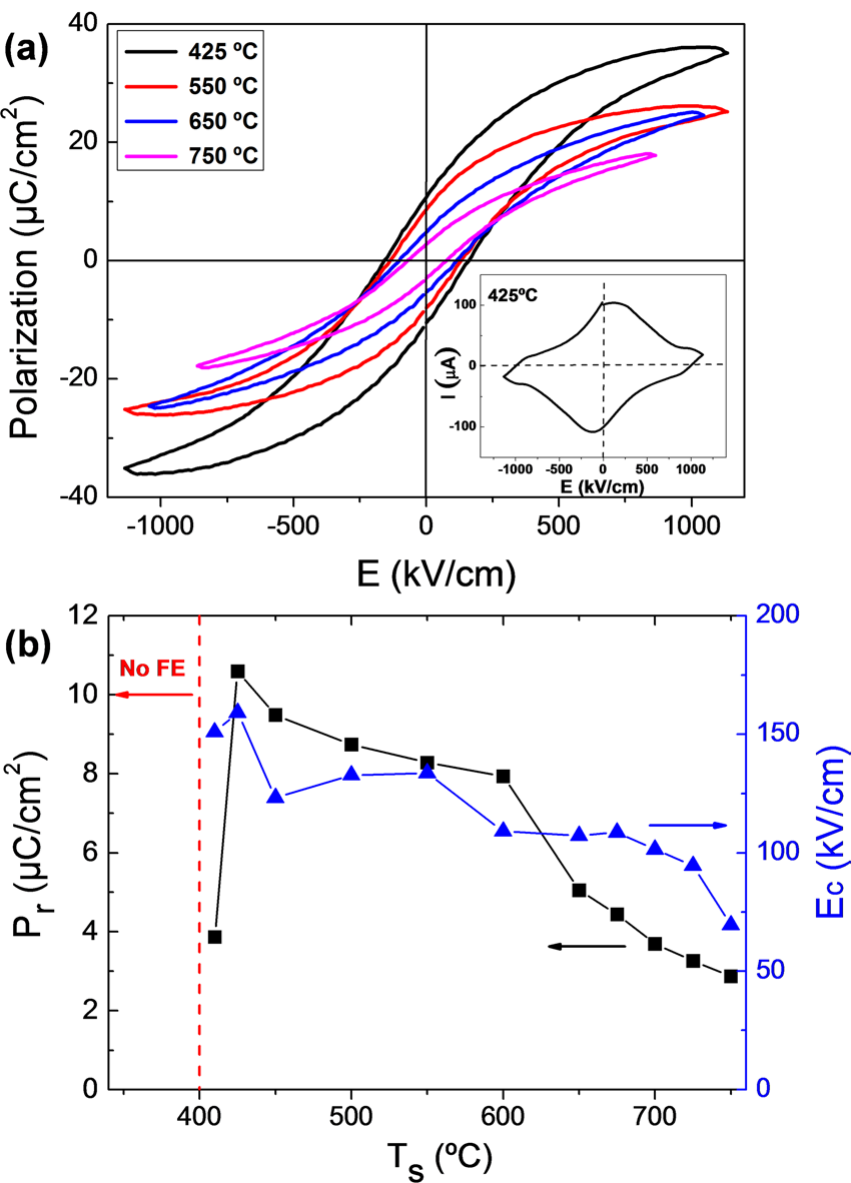
(**a**) Ferroelectric polarization loops of the T_s_ = 425 °C, 550 °C, 650 °C and 750 °C BTO films on LNO/CeO_2_/YSZ/Si(001), with the current (I) – electric field (E) curve corresponding to the T_s_ = 425 °C sample in the inset. (**b**) Remnant polarization (black squares, left axis) and coercive field (blue triangles, right axis) as a function of T_s_.

The BTO films deposited on Si at 410 °C or higher T_s_ are ferroelectric. The ferroelectric polarization loops of a selection of the films are plotted in Fig. 4a. The loops were obtained from current - electric field measurement (the corresponding to the T_s_ = 425 °C sample is presented in the inset). The remnant polarization P_r_ and electrical coercive field E_c_ of the films range within 3–11 μC/cm^2^ and 70–160 kV/cm, respectively. The films deposited at the lowest temperatures, 375 and 400 °C, did not display current ferroelectric switching peaks. Figure 4b presents the values of remnant polarization and electrical coercive fields of the BTO films on Si as a function of the deposition temperature. The threshold for ferroelectric behavior is T_s_ = 410 °C, and for higher T_s_ the remnant polarization increases sharply to a maximum value of around 11 μC/cm^2^ (T_s_ = 425 °C). For higher deposition temperatures, P_r_ decreases monotonically with T_s_. In the case of the coercive field the dependence is similar, with a reduction from 160 kV/cm (T_s_ = 425 °C) to 70 kV/cm (T_s_ = 750 °C).

**Figure 5:**
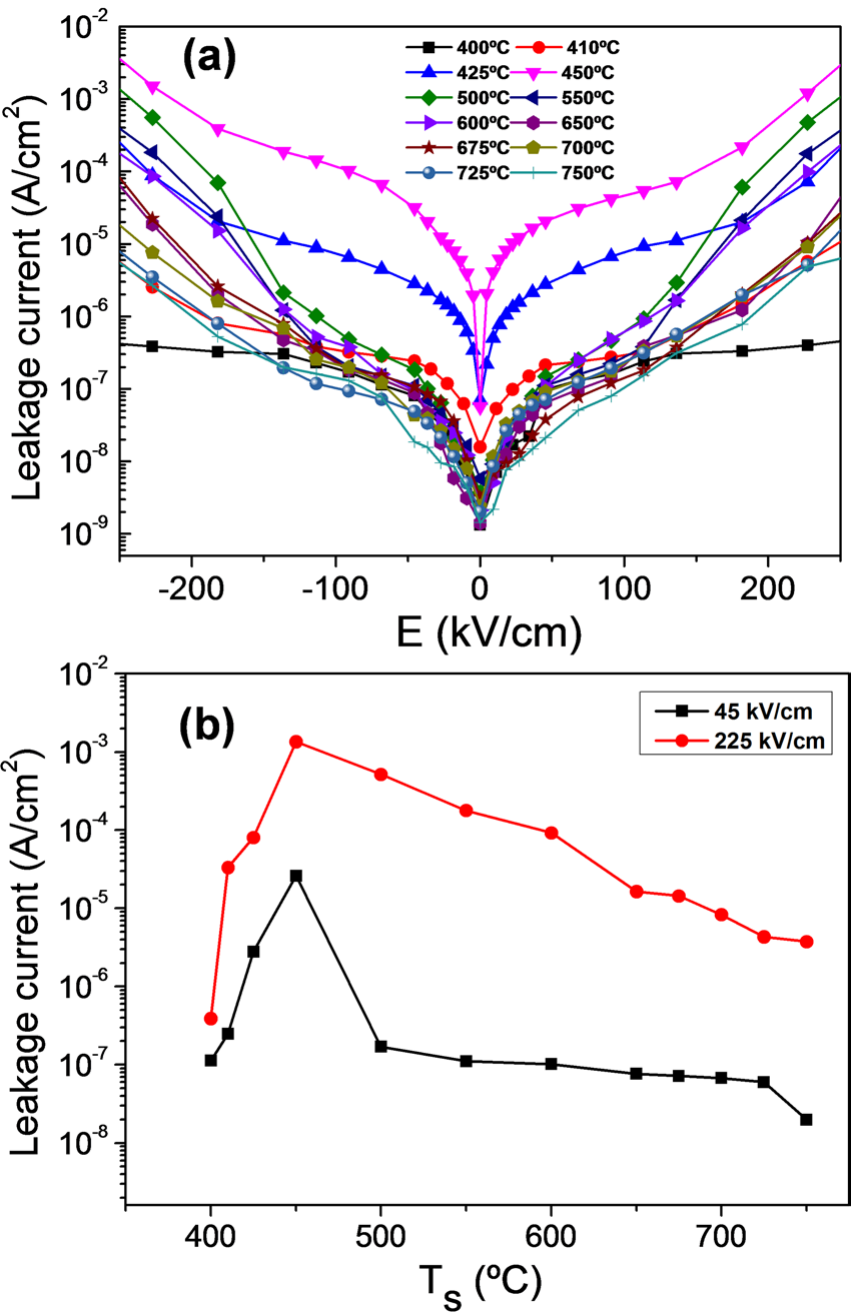
(**a**) Leakage current curves of the BTO films on LNO/CeO_2_/YSZ/Si(001). (**b**) Leakage current at 45 kV/cm (black squares) and 225 kV/cm (red circles) as a function of the deposition temperature.

The leakage curves of the series of BTO films on Si are shown in Fig. 5a, and the values of leakage current at 45 and 225 kV/cm are plotted as a function of T_s_ in Fig. 5b. The leakage current depends notably on T_s_, particularly in the samples deposited at low temperatures. The T_s_ = 375 °C sample was highly insulating and the small area of the contacts did not permit reliable measurements. The conductivity increases sharply with T_s_, the T_s_ = 450 °C sample being the most conductive of the series. In particular, the leakage current at 45 kV/cm increases from 10^−7^ A/cm^2^ (T_s_ = 400 °C) to around 3 × 10^−5^ A/cm^2^ (T_s_ = 450 °C), and from 4 × 10^−7^ A/cm^2^ to 10^−3^ A/cm^2^ at 225 kV/cm. Films deposited above T_s_ = 450 °C are more insulating, with a monotonic reduction of the leakage current with Ts, presenting the T_s_ = 750 °C samples leakage current below 1 × 10^−7^ A/cm^2^ and 4 × 10^−6^ A/cm^2^ at 45 and 225 kV/cm, respectively. We emphasize that the leakage of these films is comparable to state of the art BTO films on perovskite single crystalline substrates^5,7^ or to thick epitaxial Pb(Zr,Ti)O_3_ films on Si(001)^18^.

**Figure 6:**
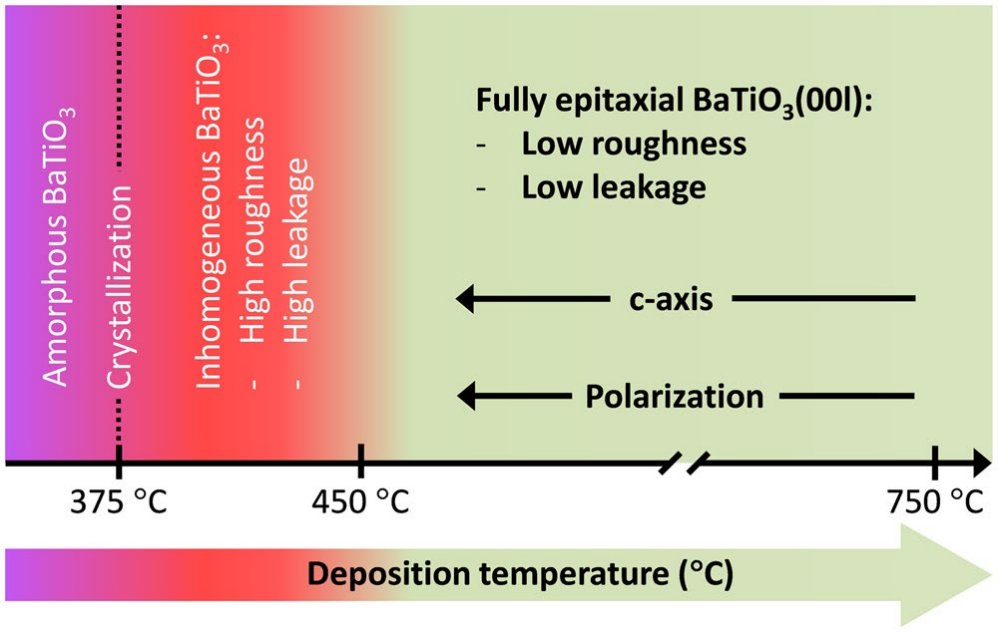
Schematics of the influence of the deposition temperature on the crystallinity and properties of BaTiO_3_ thin films on LNO/CeO_2_/YSZ/Si(001).

The impact of the deposition temperature on the properties of BTO is schematized in Fig. 6. Above the crystallization threshold at 375 °C there is a temperature window around 75 °C wide, up to T_s_ ~ 450 °C where films have a large strain and ferroelectric polarization, but are rough and present high leakage. XRD and AFM characterization point to in-homogeneous crystallinity as the cause. From T_s_ ~ 450 °C to ~750 °C, the films are very flat and are highly insulating. In this temperature window, about 300 °C wide, the BTO films are c-oriented and show a monotonic variation of the *c*-axis parameter and the ferroelectric polarization. The unit cell of BTO is highly expanded, likely due to the presence of point defects^19–22^. The influence of the deposition temperature on these defects permits the control of the *c*-axis parameter and the ferroelectric polarization.

**Figure 7:**
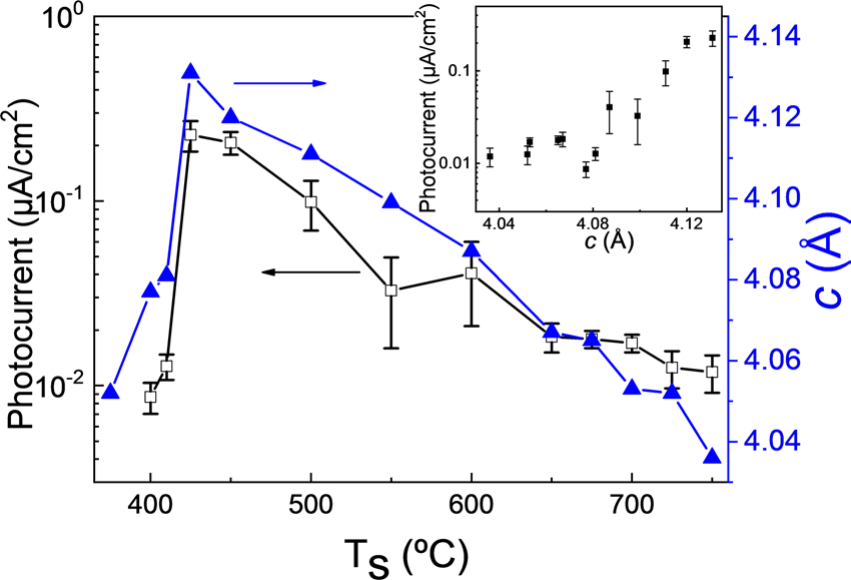
Dependence with the deposition temperature of the photocurrent (left axis, open black squares) and the out-of-plane lattice parameter (right axis, solid blue triangles). Photocurrent was measured in at least ten pairs of contacts in each film. The vertical lines in the photocurrent data indicate the ranges of values measured. Inset: photocurrent against out-of-plane lattice parameter.

In contrast to extended defects, the identification of randomly distributed point defects in oxide thin films by transmission electron microscopy is challenging and indirect methods are typically used^21^. Here we have used the photocurrent induced by illumination with 405 nm photons (energy of 3.06 eV, close but smaller than the optical gap of BTO as a probe for point defect concentration. Indeed, it is expected that point defects shall induce in-gap states promoting larger optical absorption of sub-bandgap photons and enlarge the photocurrent. The photocurrent increases with T_s_ from 8 × 10^−3^ μA/cm^2^ (T_s_ = 400 °C sample) up to above 0.2 μA/cm^2^ in the T_s_ = 425 °C and T_s_ = 450 °C samples, and then decreases progressively to around 0.1 μA/cm^2^ in the T_s_ = 700 °C and T_s_ = 750 °C samples (Fig. 7). The out-of-plane lattice parameter and the photocurrent display a very similar dependence on the deposition temperature. The photocurrent is higher in the more strained films (Fig. 7, inset), supporting that cell expansion is caused by the defects responsible of the photoresponse. On the other hand, other authors reported shifted (imprinted) ferroelectric polarization loops in epitaxial BTO films grown by pulsed laser deposition on perovskite substrate and suggested that the internal electric field to be related to the presence of aligned defect dipoles^8^. To discern the presence of defect dipoles in our BTO films we prepared symmetric LNO/BTO/LNO capacitors on (001)-oriented LaAlO_3_ (LAO) at T_s_ = 700 °C. Capacitors with symmetric electrodes were grown on purpose to minimize imprint due differences on screening properties and work functions of electrodes. The ferroelectric polarization loop (Supplementary Information Fig. S4) shows an imprint field (≈50 kV/cm), pointing towards the top LNO electrode. The presence of an internal electric field in symmetric capacitors is consistent with the existence of aligned defect dipoles in our BTO films.

**Figure 8:**
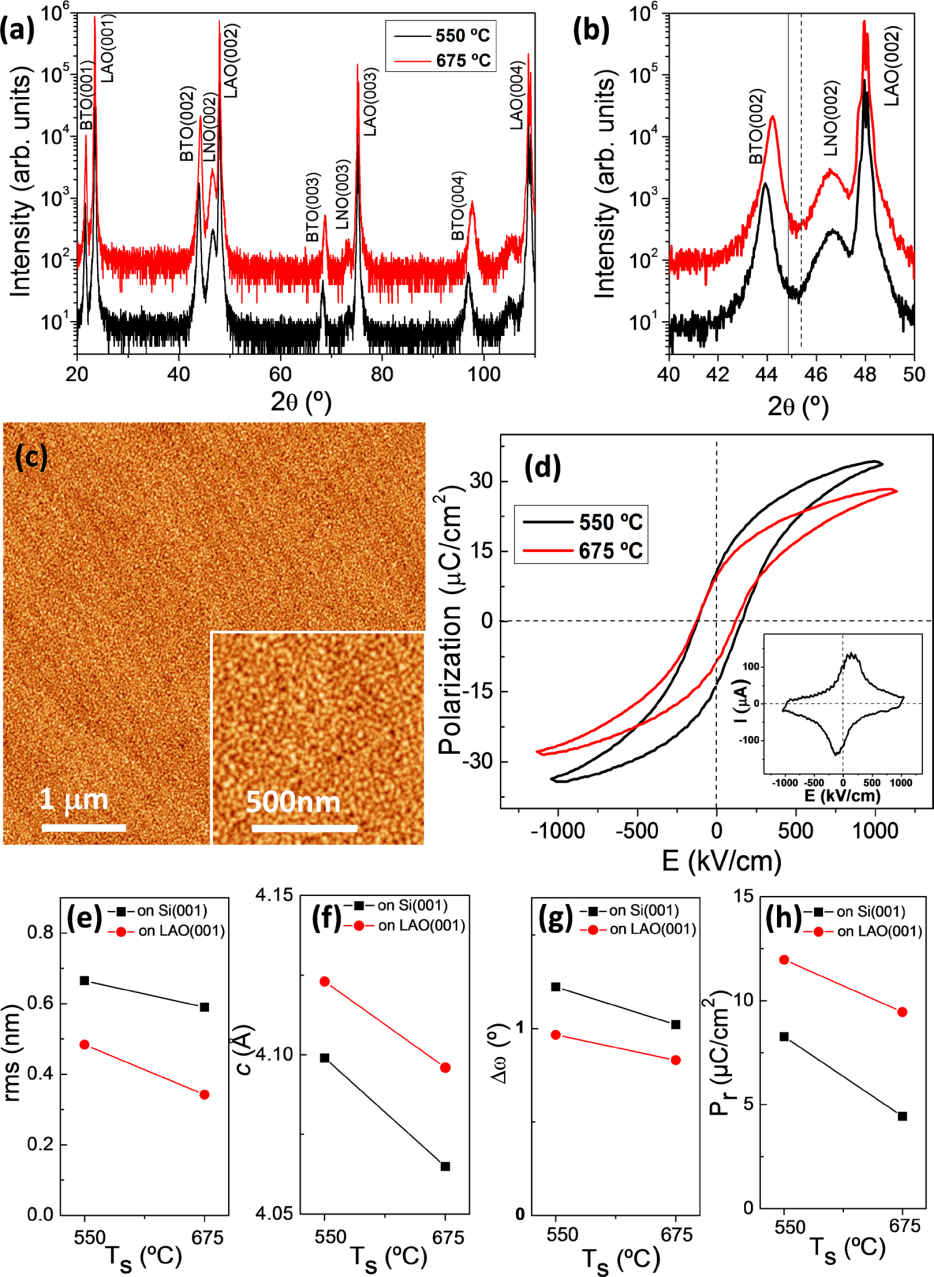
(**a**) XRD θ-2θ scans of the films deposited on LNO/LaAlO_3_(001) at T_s_ = 550 °C (black line) and T_s_ = 675 °C (red line). The intensity scale is logarithmic and the diffractograms are shifted vertically for clarity. (**b**) Zoomed region of the θ-2θ scans around the (002) reflections of BTO, LNO and LAO. The vertical solid and dashed lines mark the position of the BTO(002) and BTO(200) reflections in bulk BTO, respectively. (**c**) Topographic AFM 5 μm × 5 μm image (inset: 1 μm × 1 μm) of the film deposited at T_s_ = 675 °C (z-scale: 3 nm). (**d**) Ferroelectric polarization loops of the T_s_ = 550 °C (black line) and 675 °C films on LAO, with the current – electric field curve corresponding to the 425 °C sample in the inset. Comparison of rms roughness (**e**), out-of-plane lattice parameter (**f**), full width at half maximum of the ω-scan (**g**), and remnant polarization (**h**) of BTO films on Si (black squares) and LAO (red circles) at T_s_ = 550 °C and T_s_ = 675 °C.

It is remarkable that the control of strain and polarization by the deposition temperature has been demonstrated in thick BTO films (thicker than 100 nm) integrated epitaxially with Si(001). This is clearly the most convenient substrate for applications, and the demonstration that integration of epitaxial BTO with high polarization can deposited at temperature as low as 450 °C is also of relevance towards the integration in silicon chips. Moreover, the growth strategy here presented should also be valid for BTO films grown on single crystalline oxide substrates, which are commonly used as platforms to grow BTO films. To confirm this statement we have deposited two BTO/LNO bilayers on LAO at 550 and 675 °C. Due to the moderately small lattice mismatch (~1.3%) between the LNO electrode and the LAO substrate, the XRD specular θ-2θ scans of the bilayers (Fig. 8a) only display (00 l) reflections. The zoom of the scans around the (002) reflections shows narrow BTO peaks at positions that confirm the expected c-orientation and expanded *c*-axis, being the strain in the T_s_ = 550 °C sample (c = 4.123 Å) higher than in the T_s_ = 675 °C one (c = 4.096 Å). Reciprocal space maps around asymmetrical (103) reflections are shown in Supplementary Information Fig. S5. The LNO bottom electrode is fully strained, whereas BTO presents in-plane lattice parameter coincident with the a-axis of bulk BTO. It indicates that the films are plastically relaxed, and that the defects cause anisotropic unit-cell deformation, with expansion of the unit cell along the out-of-plane direction. Topographic AFM images show that the films are very flat, and in the case of the T_s_ = 675 °C sample rms roughness below 0.4 nm and morphology of terraces and steps can be observed in the 5 μm × 5 μm image (Fig. 8c) in spite of the very small islands (Fig. 8c, inset), usually present in BTO films^15,23,24^. The ferroelectric loops (Fig. 7d) show larger polarization in the T_s_ = 550 °C film, in agreement with its higher lattice strain. The values of rms roughness, c-axis parameter, BTO(002) rocking curve width and remnant polarization are plotted (red circles) against T_s_ (550 °C or 675 °C) in Fig. 8e–h, respectively. They include the data of the corresponding samples on Si (black squares). Obviously, the BTO films deposited on Si(001) and LAO(001) substrates display the same trend of film morphology (rms), crystallinity (*c*-axis parameter and rocking curve) and polarization.

## Discussion

In summary, *c*-oriented BTO films have been epitaxially integrated with Si(001) with tailored strain of the polar axis in a broad range from 0 to above 2%. In contrast to the usual strain engineering methodology, the selection of a particular strain is achieved without necessity of selecting a specific substrate, and it is not limited to very thin ferroelectric films. To accomplish this, using high energy pulsed laser deposition technique, the substrate temperature is used as a knob within a wide temperature window of about 300 °C to grow epitaxial BTO films with the desired c-axis strain. The ferroelectric polarization scales with the strain, and, concomitantly, it is determined by the selected deposition temperature. Using this methodology, either on Si(001) or on perovkite oxide single crystals, epitaxial BTO films with large polarization and very small roughness and electrical leakage are produced. Of technological relevance, it is demonstrated that these properties are achieved integrating BTO with Si(001) at temperatures as low as 450 °C.

## Methods

### Thin films deposition

BTO films were deposited on Si(001) substrates using a multilayer buffer. The first buffer layer, yttria-stabilized zirconia (YSZ), was grown on Si(001) without removing the native silicon oxide, and CeO_2_, LNO and BTO were sequentially deposited. The heterostructures were fabricated in a single process by pulsed laser deposition (KrF excimer laser). The buffer layers were grown using the same parameters, including substrate temperature of 800 °C for YSZ and CeO2, and 700 °C for LNO, and oxygen pressure of 4 × 10–4 mbar for YSZ and CeO_2_, and 0.15 mbar for LNO; additional experimental conditions are reported elsewhere^15,17,25^. The deposition temperature was measured using a thermocouple inserted in the middle of the heater block. The thicknesses of the BTO, LNO, CeO_2_ and YSZ layers are 110, 30, 20, and 60 nm, respectively. BTO films were deposited under a dynamic oxygen pressure of 0.02 mbar, being the laser frequency of 5 Hz. A series of 13 samples was prepared varying the substrate temperature in steps of 50 °C between 400 and 750 °C, and additional samples were deposited at T_s_ = 375, 410, 425, 675 and 725 °C. Moreover, two BTO/LNO bilayers were prepared on LAO (001) at 550 and 675 °C. After deposition, the BTO films were cooled down to room temperature under an oxygen atmosphere of 0.2 mbar. Two additional films deposited on Si(001) at T_s_ = 700 °C were cooled down under 200 mbar of oxygen, adding for one of an *in-situ* at 600 °C for 1 hour. The effect of *ex-situ* annealing (1 hour, 200 mbar) was investigated on the T_s_ = 450 °C and 600 °C films on Si(001). Both samples were annealed sequentially two times at 450 °C and 600 °C for 1 hour under 200 mbar.

### Structural and electrical characterization

The crystal structure was characterized by X-ray diffraction (XRD), determining the out-of-plane lattice parameter of BTO from symmetrical θ-2θ scans (Cu Kα radiation), and measuring rocking curves of the BTO(002) reflection. Reciprocal space maps around BTO(203) and Si(224) of selected samples were measured by high resolution XRD using Cu Kα_1_ radiation to obtain the in-plane lattice parameter of BTO, and ϕ-scans around BTO(110), LNO(110), YSZ(220) and Si(220) were performed to determine the epitaxial relationships. The surface morphology of BTO was characterized by atomic force microscopy (AFM) in dynamic mode. Platinum top electrodes, 20 nm thick and 60 μm × 60 μm in size, were deposited using dc magnetron sputtering through stencil masks. Ferroelectric polarization loops and leakage current were measured at room temperature in top-top configuration^26^ (two BTO capacitors were measured in series, contacting two top Pt electrodes and using the conducting LNO buffer layer as common bottom electrode) by means of an AixACCT TFAnalyser2000 platform. Ferroelectrics loops were obtained by sweeping an electric field at a constant rate with a frequency of 10 kHz and measuring the current, using the dielectric leakage current compensation (DLCC) to minimize leakage current effects^27^. Leakage current curves were measured using the TFAnalyser2000 platform (or an electrometer for the samples grown at T_s_ = 410 °C or below, which are highly insulating), using 3 s integration time, and averaging I-V curves increasing and decreasing the voltage. Short-circuit photocurrent was measured by illuminating the sample with blue laser of wavelength 405 nm (Shenzhen 91 Laser Co.). Photoinduced current was monitored as a function of time (t) switching on the illumination at 5 s and off at 15 s. The spot diameter was of 200 μm safely illuminating the measured electrode.

## Electronic supplementary material

Supplementary information file
