# Supplementary material for: Tailoring Lattice Strain and Ferroelectric Polarization of Epitaxial BaTiO3 Thin Films on Si(001)
Source: Sci Rep. 2018 Jan 11;8:495. doi: 10.1038/s41598-017-18842-5 (PMC5765027; doi:10.1038/s41598-017-18842-5)
Supplement: Supplementary file 1 — Supplementary information file [file 41598_2017_18842_MOESM1_ESM.pdf]

Tailoring Lattice Strain and Ferroelectric Polarization of Epitaxial $BaTiO_3$ Thin Films on Si(001)

Jike Lyu, Ignasi Fina, Raúl Solanas, Josep Fontcuberta & Florencio Sánchez

Institut de Ciència de Materials de Barcelona (ICMAB-CSIC), Campus UAB, Bellaterra 08193, Barcelona, Spain

Supplementary Information

Supplementary Information S1: XRD reciprocal space maps of BTO films on Si(001)

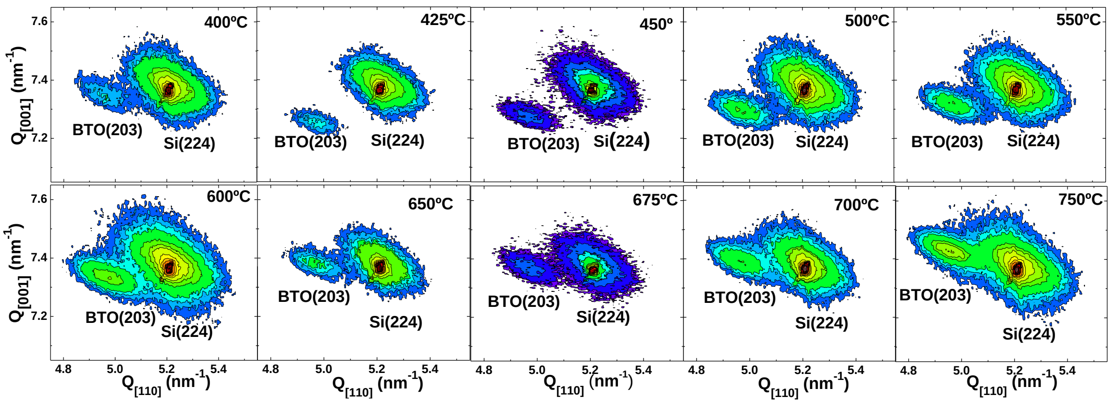

Figure S1: XRD reciprocal space maps (RSM) of BTO films on Si(001) deposited at various substrate temperature T_s (indicated in the top right side of each panel). The mapped reciprocal space contains asymmetrical BTO(203) and Si(224) reflections. The RSM we recorded using Cu Kα_1 using same acquisition conditions(except for the T_s = 450 °C and 675 °C samples, recorded with shorter acquisition time).

Supplementary Information S2:

Influence on strain and polarization of high oxygen pressure during cooling down and of in-situ annealing

The films reported in the manuscript were cooled down to room temperature under the same conditions. An oxygen pressure of 0.2 mbar was introduced immediately after the growth, and the substrate heater power was switched off. Two additional BTO/LNO/CeO_2/YSZ/Si(001) samples, deposited at T_s = 700 °C, were cooled down under a different process after the growth. Sample (1): the oxygen pressure introduced at the end of the growth was 200 mbar, and the film was cooled down to room temperature under this pressure and with the substrate heater power switched off. Sample (2): the oxygen pressure introduced at the end of the growth was 200 mbar, and it was cooled to room temperature after a dwell time of 1 hour at 600 °C. The two different cooling down processes do not cause great effects on the BTO strain and

polarization (Figure S2) respect to the reference sample cooled down under 0.2 mbar of oxygen (out-of-plane lattice parameter c = 4.053 Å, remnant polarization P_r = 3.7 μC/cm^2. In the case of Sample 1 (cooled down under 200 mbar), c and P_r are 4.049 Å and 3.1 μC/cm^2, respectively, and in sample 2 (cooled down under 200 mbar, and in-situ annealing at 600 °C for 1 hour) c and P_r are 4.043 Å and 3.3 μC/cm^2, respectively. Figures S2a and S2b show, respectively, the T_s dependence of the c-axis values and P_r values, and the corresponding values for samples (1) and (2) are plotted (blue squares). The P_r values of the series is plotted against c in Figure S2c (data corresponding to samples (1) and (2) are plotted as blue squares). There is a clear linear dependence, and samples (1) and (2) scale well in the series, suggesting a slight reduction in the amount of defects in the two samples.

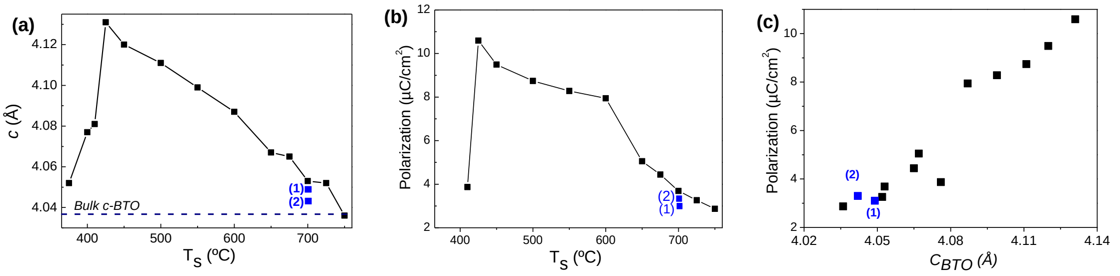

Figure S2: Dependence with the deposition temperature T_s of BTO on LNO/CeO_2/YSZ/Si(001) of (a) out-of-plane lattice parameter c, and (b) remnant polarization. (c) Remnant polarization plotted against T_s. Black squares correspond to the samples discussed in the manuscript (they were cooled down to room temperature under an oxygen pressure of 0.2 mbar introduced immediately after the growth). Samples labelled 1 and 2 (blue squares) were cooled down under a different procedure. Sample (1): the oxygen pressure introduced at the end of the growth was 200 mbar, and it was cooled down to room temperature under this pressure. Sample (2): the oxygen pressure introduced at the end of the growth was 200 mbar, and it was cooled to room temperature under this pressure after a dwell time of 1 hour at 600 °C.

Supplementary Information S3:

Influence on strain of ex-situ annealing

The T_s = 450 °C and 600 °C films on Si(001) were cut in two pieces, and only one of them was used for two sequential annealings. were annealed during 1 hour under 200 mbar of atomic oxygen. The cut piece of each sample was measured by XRD (θ-2θ scan), annealed at 450 °C during 1 hour under 200 mbar of atomic oxygen, measured by XRD, annealed at 600 °C during 1 hour under 200 mbar of atomic oxygen, and finally measured by XRD.

Figure S3 shows the θ-2θ scans (a). The samples show almost negligible differences after annealing at 450 °C. The annealing at 600 °C has very small effect on the T_s = 600 °C sample, and in the T_s = 450 °C sample there is a small decrease of the out-of-plane parameter. The variation of the lattice parameters with the annealings is plotted in (b). (c) Dependence of the out-of-plane lattice parameter with the deposition temperature T_s, including the data corresponding to the *ex-situ* annealed samples (open triangles) and the *in-situ* annealed samples (open squares).

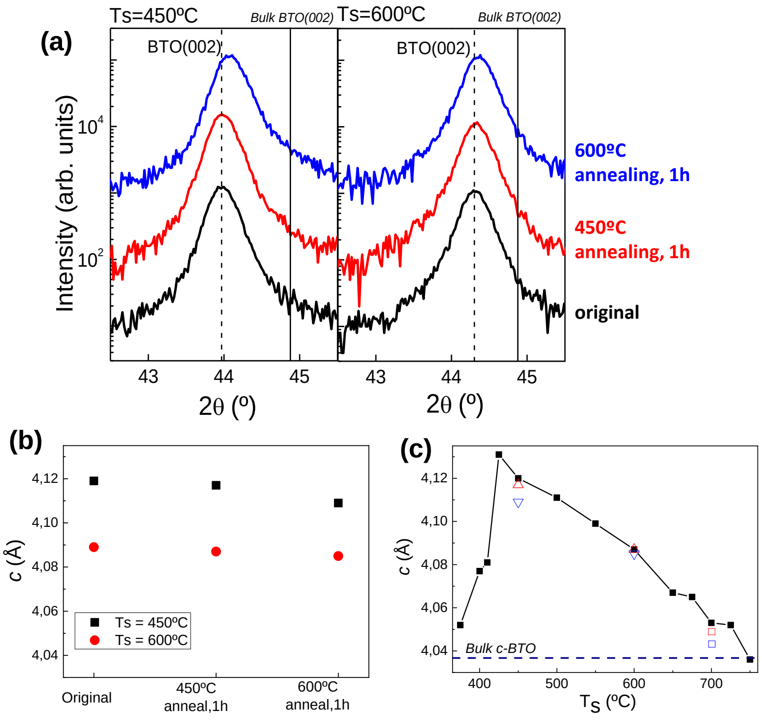

Figure S3: XRD θ-2θ scan of the (a) T_s = 450 °C (left panel) and 600 °C (right panel) films on Si(001) as-deposited (black curves), after annealing at 450 °C (red curves), and after 600 °C (blue curves). The patterns are shifted vertically for clarity. The vertical solid line mark the position of the BTO(002) reflection in bulk BTO, and the vertical dashed line mark the corresponding position of the BTO(002) reflections of the as-deposited films. (b) Out-of-plane lattice parameter c of the as-deposited and annealed films. (c) Dependence of the out-of-plane lattice parameter c with the deposition temperature T_s of BTO on LNO/CeO_2/YSZ/Si(001), including the values of the T_s = 450 °C and T_s = 600 °C samples after the *ex-situ* first annealing at 450 °C (open red triangles up) and second annealing at 600 °C (open blue triangles down). The corresponding data of the *in-situ* annealed T_s = 700 °C samples are plotted (open red and blue squares).

Supplementary Information S4: Polarization loops of symmetric on $LaNiO_3$/$BaTiO_3$/$LaNiO_3$ capacitors on $LaAlO_3$(001)

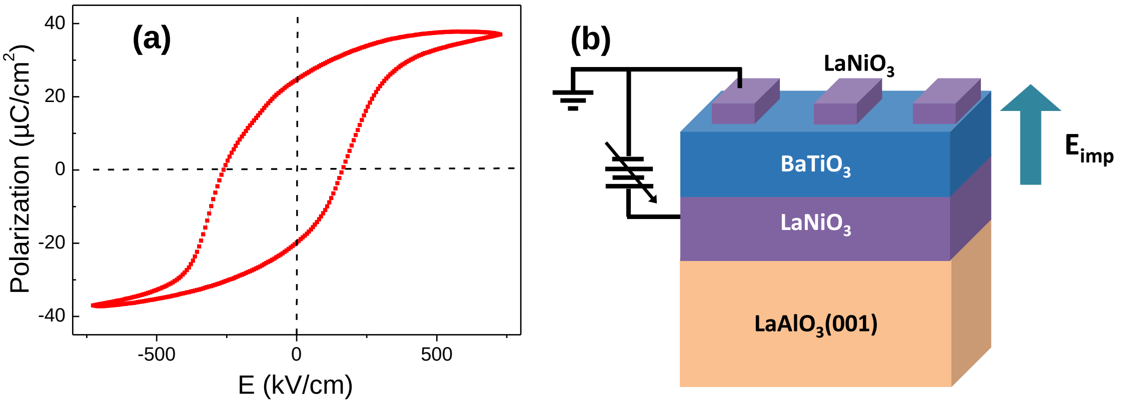

Figure S4: (a) Polarization ferroelectric loop (DLCC measurement, 1 kHz, circular top electrode of diameter 200 μm) of a symmetric $LaNiO_3/BaTiO_3/LaNiO_3$ capacitor on $LaAlO_3$(001). A sketch is plotted in (b). The BTO film was deposited at 700 °C. The top LNO electrode was deposited by pulsed laser deposition at 700 °C and 0.15 mbar of oxygen, using a steel shadow mask in contact with the BTO film. The ferroelectric loop was measured applying positive voltage to bottom LNO electrode and grounding a top LNO electrode. The observed horizontal negative shift corresponds to an imprint field of ≈50 kV/cm, which is directed from the bottom electrode towards the top electrode.

Supplementary Information S5: XRD reciprocal space maps of BTO films on $LaAlO_3$(001)

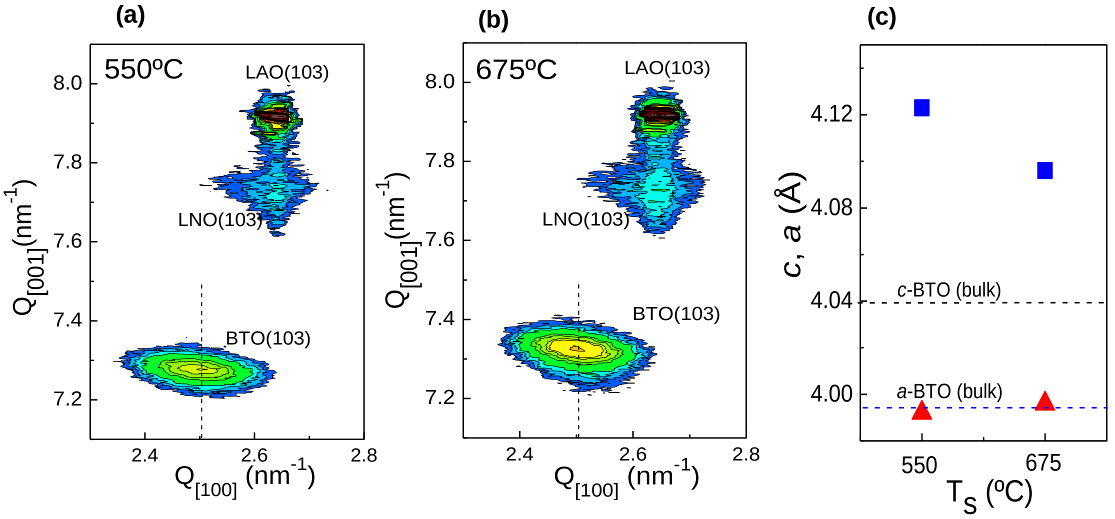

Figure S5: XRD reciprocal space maps (RSM) of BTO films deposited on LNO/$LaAlO_3$(001) at T_s = 550 °C (a) and T_s = 675 °C (b). The mapped reciprocal space contains asymmetrical (103) reflections of BTO, LNO and LAO. The RSMs we recorded using Cu Kα_1. The vertical dashed line indicates the $Q_{[100]}$ coordinate for bulk BTO (a-axis). (c) Out-of-plane (blue squares) and in-plane (red triangles) lattice parameters of the two BTO films. The horizontal dashed lines indicate the corresponding parameters for bulk BTO.
